# Analysis of the association between the neutrophil-to-lymphocyte ratio (NLR) and liver injury complicating acute pancreatitis: a retrospective study

**DOI:** 10.3389/fmed.2026.1864881

**Published:** 2026-07-21

**Authors:** Jinbao Li, Xueling Liu, Rongzhen Zhao, Haoran Zhu, Juan Wu, Weijian Huang, Bo Li, Qinghua Wang

**Affiliations:** 1 School of Nursing, Shandong Medical and Pharmaceutical University, Binzhou, Shandong, China; 2 Department of Critical Care Medicine, Zibo Hospital of Traditional Chinese Medicine, Zibo, Shandong, China; 3 Department of Nursing, Yantai Yuhuangding Hospital, Yantai, Shandong, China; 4 Shandong Medical and Pharmaceutical University Hospital, Binzhou, Shandong, China

**Keywords:** acute liver injury, acute pancreatitis, neutrophil-to-lymphocyte ratio, predictive value, threshold effect

## Abstract

**Background:**

Acute pancreatitis is frequently complicated by acute liver injury (ALI), which exacerbates systemic inflammation and increases mortality. Early identification of high-risk patients remains challenging. The neutrophil-to-lymphocyte ratio (NLR) is a simple marker of systemic inflammation, but its association with ALI in acute pancreatitis is unclear.

**Objectives:**

To investigate the association between NLR and ALI in acute pancreatitis and evaluate its predictive value.

**Methods:**

Clinical data from 581 acute pancreatitis inpatients (2021–2025) were retrospectively analyzed. Multivariate logistic regression, generalized additive models, subgroup analyses, interaction tests, and ROC curves were used.

**Results:**

ALI occurred in 37.7% of patients. After adjustment, each one-unit NLR increase raised ALI risk by 11% (OR = 1.11, 95% CI: 1.08–1.14, *P* < 0.001). The highest NLR quartile had a 5.42-fold higher risk than the lowest. A non-linear threshold was identified at NLR = 2.92: risk rose sharply below this point (OR = 6.55) and more gradually above it (OR = 1.07), suggesting that even a mild NLR elevation may signal early liver injury risk. NLR alone predicted ALI with AUC = 0.688 (cutoff 8.26, sensitivity 59.4%, specificity 68.0%). Adding CRP and gallstones improved AUC to 0.756 (sensitivity 65.8%, specificity 76.2%), indicating that combining inflammatory markers with etiological factors enhances predictive accuracy.

**Conclusion:**

NLR independently predicts ALI in acute pancreatitis with a non-linear threshold effect. NLR ≥ 2.92 can serve as an early warning signal to initiate enhanced liver function monitoring, while NLR ≥ 8.26 identifies patients at high risk. Combining NLR with other readily available indicators significantly improves predictive performance and may facilitate early clinical decision-making.

## 1 Introduction

Acute pancreatitis (AP) is a common clinical emergency characterized by abdominal pain, primarily triggered by factors such as biliary tract disease, hyperlipidemia, and excessive alcohol consumption (1). The condition progresses rapidly and poses a serious threat to patients’ lives (2), The liver is one of the most commonly affected target organs during the course of AP. Acute liver injury (ALI), a common complication of AP, further exacerbates the systemic inflammatory response, prolongs hospital stays, and significantly increases the risk of severe illness and death (3). Clinical data indicate that the severity of ALI is closely associated with the incidence of systemic inflammatory response syndrome and multiple organ dysfunction syndrome, directly impacting the long-term prognosis of patients with AP (4–6). Approximately 20% of AP patients progress to severe acute pancreatitis, with an overall mortality rate as high as 20%–40% (7), and patients with concomitant ALI account for a substantial proportion of this high-risk group. Therefore, early warning and early intervention for AP-associated ALI are critical to reducing the incidence of severe AP and mortality rates, as well as improving patient outcomes.

However, current clinical management still has significant shortcomings. Traditional liver function markers can only diagnose liver injury after it has occurred, resulting in a considerable delay in warning and failing to meet the needs of early prevention and control. The development of a convenient, accurate, and bedside-usable biomarker for the rapid screening of ALI is of great clinical utility. The neutrophil-to-lymphocyte ratio (NLR) is a classic inflammatory marker derived from routine complete blood counts. It is rapid and inexpensive to measure and effectively reflects the body’s inflammatory activation and immune imbalance. NLR is now widely used in the assessment of various organ injuries and infectious diseases (8–10). In recent years, its value in the assessment of liver injury has gradually garnered attention. Multiple studies have confirmed a significant association between NLR and hepatocyte injury, abnormal liver function, and adverse outcomes in liver disease (11–13). Previous research has also established that NLR can be used to assess the severity of AP, predict complications, and forecast poor prognosis (14–16).

Nevertheless, existing studies exhibit significant bias, with most focusing on overall AP severity and systemic multi-organ failure. Systematic research specifically targeting AP complicated by ALI remains extremely limited. Key scientific questions—whether NLR is independently associated with AP-related ALI, whether a non-linear threshold effect exists between the two, and what warning thresholds are suitable for clinical implementation—currently lack support from large-sample data and have not yielded unified conclusions. Given these clinical challenges and research gaps, the present study focused on AP complicated by ALI as its core objective. We retrospectively collected data from inpatients with AP at a tertiary Grade A hospital in Shandong Province to conduct an in-depth analysis of the association between admission NLR levels and ALI, explore the threshold relationship between the two, and evaluate the predictive value of NLR and combined indicators for ALI. This study aims to clarify the potential of NLR in the early warning of AP-associated liver injury and to provide objective, feasible laboratory evidence for the targeted implementation of liver function monitoring and hepatoprotective therapy in clinical practice.

## 2 Methods and analysis

### 2.1 Design

This study collected clinical data from 581 patients with acute pancreatitis (AP) at a tertiary Grade A hospital in Shandong Province between December 2021 and December 2025. (1) Inclusion criteria: all patients met the 2025 revised diagnostic criteria for acute pancreatitis established by the International Association of Pancreatology (IAP) (17), satisfying at least two of the following three criteria: ① acute onset of persistent severe upper abdominal pain; ② serum lipase or amylase levels ≥ 3 times the upper limit of normal; or ③ characteristic changes of acute pancreatitis on contrast-enhanced CT, MRI, or ultrasound. (2) Exclusion criteria: ① patients with concurrent malignant tumors or chronic wasting diseases (e.g., tuberculosis, chronic hepatitis); ② patients aged < 18 years; and ③ individuals with suspected exposure to factors causing acute liver injury within 7 days prior to admission (e.g., use of hepatotoxic medications). (3) Liver injury was defined as the occurrence during hospitalization of any of the following five criteria: ① serum alanine aminotransferase (ALT) > 3 × upper limit of normal (ULN); ② serum aspartate aminotransferase (AST) > 3 × ULN; ③ γ-glutamyltransferase (GGT) > 2 × ULN; ④ alkaline phosphatase (ALP) > 2 × ULN; or ⑤ total bilirubin (TBIL) > 2 × ULN (18).

### 2.2 Data collection and definition

The NLR was calculated using the formula: NLR = absolute neutrophil count/absolute lymphocyte count (19). The collected data included general demographic characteristics (age and sex); lifestyle factors (smoking history and alcohol consumption history); laboratory test results, including lipid profiles [total cholesterol (TC), triglycerides (TG), and low-density lipoprotein cholesterol (LDL-C)], inflammatory markers [white blood cell count (WBC), lymphocyte count (LYM), monocyte count (MONO), C-reactive protein (CRP), and platelet count (PLT)], and other parameters [albumin (ALB)]; as well as comorbidities (diabetes, gallstones, hyperlipidemia, and ascites).

### 2.3 Statistical analysis

Continuous variables were expressed as mean ± standard deviation (SD) if normally distributed, or as median with interquartile range (IQR) if skewed; categorical variables were expressed as frequencies (percentages). Participants were stratified according to NLR quartiles, and baseline characteristics were compared across groups using analysis of variance (ANOVA) or the Kruskal-Wallis test for continuous variables, and the chi-square test for categorical variables. Multivariate logistic regression analysis was used to examine the association between NLR and liver injury. Three regression models were constructed: Model 1 was unadjusted for any confounders; Models 2 and 3 were adjusted for age, sex, diabetes, gallstones, alcohol consumption history, smoking history, and hyperlipidemia. A linear trend test was performed to assess the consistency of the association. A generalized additive model (GAM) with smoothed curve fitting was employed to explore whether a non-linear relationship existed between NLR and liver injury. If a non-linear relationship was identified, a recursive algorithm was used to determine significant inflection points in the association, and threshold effect analysis was conducted to compare the logistic regression model with the two-stage logistic regression model. Subgroup analyses and interaction tests were performed for age, sex, diabetes, gallstones, smoking history, alcohol consumption history, and ascites, with adjustment for corresponding confounders. A non-significant interaction *P*-value indicated that the association between NLR and liver injury was stable across subgroups, whereas a significant interaction *P*-value suggested heterogeneity, warranting further analysis of subgroup-specific effects. All statistical analyses were performed using SPSS 29.0 and R 4.5.1. A *P*-value < 0.05 was considered statistically significant.

## 3 Results

### 3.1 Baseline characteristics

This study included a total of 581 patients with acute pancreatitis (AP), of whom 219 developed acute liver injury (ALI) during hospitalization, yielding a prevalence of 37.7%. Compared with the non-ALI group, patients in the ALI group had a higher proportion of males, a slightly higher proportion of patients with a history of alcohol consumption, and a significantly higher prevalence of gallstones; all differences were statistically significant (*P* < 0.05). In addition, patients in the ALI group exhibited a more severe inflammatory response, as reflected by significantly higher levels of C-reactive protein, neutrophil count, total cholesterol, triglycerides, NLR, and D-dimer, as well as significantly lower levels of lymphocyte count, albumin, and platelet count (*P* < 0.01). The proportion of patients with diabetes was significantly lower in the ALI group, while ascites was more prevalent among patients with liver injury (Table 1).

**TABLE 1:** Baseline characteristics of acute pancreatitis patients with and without liver injury.

Variables	Subgroups	Total, *N* = 581, *n* (%)	non-ALI N = 362, *n* (%)	ALI *N* = 219, *n* (%)	*P*-value
Age, *n* (%)	<60 years	64 (11.0)	35 (9.7)	29 (13.2)	0.231
	≥60 years	517 (89.0)	327 (90.3)	190 (86.8)	
Gender, *n* (%)	Female	183 (31.5)	125 (34.5)	58 (26.5)	0.053
	Male	398 (68.5)	237 (65.5)	161 (73.5)	
HTG, *n* (%)	No	371 (63.9)	211 (58.3)	160 (73.1)	<0.001
	Yes	210 (36.1)	151 (41.7)	59 (26.9)	
Diabetes, *n* (%)	No	415 (71.4)	245 (67.7)	170 (77.6)	0.013
	Yes	166 (28.6)	117 (32.3)	49 (22.4)	
Ascites, *n* (%)	No	391 (67.3)	258 (71.3)	133 (60.7)	0.011
	Yes	190 (32.7)	104 (28.7)	86 (39.3)	
Gallstones, *n* (%)	No	405 (69.7)	294 (81.2)	111 (50.7)	<0.001
	Yes	176 (30.3)	68 (18.8)	108 (49.3)	
Drink, *n* (%)	No	422 (72.6)	267 (73.8)	155 (70.8)	0.493
	Yes	159 (27.4)	95 (26.2)	64 (29.2)	
Smoke, *n* (%)	No	329 (56.6)	190 (52.5)	139 (63.5)	0.012
	Yes	252 (43.4)	172 (47.5)	80 (36.5)	
BMI	–	25.1 (24.2, 28.37)	25.6 (24.33, 28.75)	25.0 (23.8, 27.36)	0.004
ALB, g/L	–	42.70 (38.30, 45.40)	43.10 (39.05, 45.58)	41.70 (36.95, 44.95)	0.004
CRP, mg/L	–	29.00 (5.40, 85.80)	16.95 (4.50, 65.02)	56.10 (7.28, 126.25)	<0.001
TC, mmol/L	–	5.51 (4.30, 7.40)	5.09 (4.04, 6.96)	5.71 (4.44, 7.55)	0.008
LDL, mmol/L	–	2.90 (2.21, 3.53)	2.90 (2.25, 3.52)	2.90 (2.18, 3.55)	0.856
TG, mmol/L	–	3.31 (1.17, 15.53)	2.06 (1.08, 7.72)	4.33 (1.23, 17.37)	0.001
WBC, 10^9^/L	–	11.0 (8.4, 13.7)	10.6 (8.2, 13.1)	11.7 (8.7, 14.5)	0.005
PLT, 10^9^/L	–	229 (187, 278)	237.5 (194.25, 281)	219 (173, 273)	0.008
HCT, %	–	42.0 (38.0, 45.00)	42.0 (39.0, 45.0)	42.0 (38.0, 45.0)	0.965
LYM, 10^9^/L	–	1.30 (0.80, 1.80)	1.40 (1.00, 2.00)	1.00 (0.62, 1.50)	<0.001
MONO, 10^9^/L	–	0.5 (0.4, 0.8)	0.56 (0.4, 0.8)	0.5 (0.4, 0.8)	0.711
Neut, 10^9^/L	–	8.7 (6.10, 11.60)	8.60 (5.43, 10.60)	9.3 (7.05, 12.5)	<0.001
NLR	–	6.84 (3.64, 11.79)	5.47 (3.01, 9.32)	9.34 (5.15, 16.26)	<0.001
RDW, fL	–	40.5 (38.4, 42.2)	40.3 (38.23, 41.48)	40.6 (38.85, 43.0)	<0.001
D-D, mg/L	–	0.67 (0.28, 1.74)	0.52 (0.23, 1.24)	1.07 (0.48, 2.17)	<0.001

BMI, body mass index; HTG, hypertriglyceridemia; ALB, albumin; CRP, C-reactive protein; TC, total cholesterol; HDL, high-density lipoprotein cholesterol; LDL, low-density lipoprotein cholesterol; TG, triglyceride; WBC, white blood cell; PLT, platelet; HCT, hematocrit; LYM, lymphocyte; MONO, monocyte count; Neut, neutrophil; NLR, neutrophil-to-lymphocyte ratio; RDW, red blood cell distribution width; D-D, D-dimer.

Based on the NLR distribution, the study subjects were divided into four quartiles: first quartile (NLR ≤ 3.64, *n* = 146), second quartile (3.64 < NLR ≤ 6.84, *n* = 145), third quartile (6.84 < NLR ≤ 11.78, *n* = 145), and fourth quartile (NLR > 11.78, *n* = 145). With increasing NLR quartile, levels of total cholesterol, triglycerides, D-dimer, white blood cell count, neutrophil count, monocyte count, and C-reactive protein (CRP) gradually increased, whereas lymphocyte count gradually decreased. Notably, the fourth quartile had a higher proportion of patients with both gallstones and ascites. Detailed comparisons of indicators across the four groups are presented in Table 2.

**TABLE 2:** Baseline characteristics of acute pancreatitis patients according to neutrophil-to-lymphocyte ratio (NLR) quartiles.

Variables	Subgroups	Total (*N* = 581)	Q1 (*N* = 146)	Q2 (*N* = 145)	Q3 (*N* = 145)	Q4 (*N* = 145)	*P*-value
NLR	–	6.84 (3.64, 11.79)	2.58 (1.85, 3.07)	5.05 (4.35, 5.85)	8.80 (8.03, 9.89)	17.4 (14.5, 22.0)	<0.001
Age (%)	<60 years	64 (11.0)	16 (11.0)	18 (12.4)	19 (13.1)	11 (7.6)	0.445
	≥60 years	517 (89.0)	130 (89.0)	127 (87.6)	126 (86.9)	134 (92.4)	
Gender, *n* (%)	Female	183 (31.5)	52 (35.6)	37 (25.5)	44 (30.3)	50 (34.5)	0.237
	Male	398 (68.5)	94 (64.4)	108 (74.5)	101 (69.7)	95 (65.5)	
BMI	–	25.1 (24.2, 28.37)	25.0 (23.01, 28.0)	25.71 (24.49, 28.34)	25.0 (24.4, 28.73)	25.23 (24.33, 28.25)	0.13
HTG, *n* (%)	No	371 (63.9)	104 (71.2)	89 (61.4)	88 (60.7)	90 (62.1)	0.199
	Yes	210 (36.1)	42 (28.8)	56 (38.6)	57 (39.3)	55 (37.9)	
Diabetes, *n* (%)	No	415 (71.4)	104 (71.2)	106 (73.1)	106 (73.1)	99 (68.3)	0.775
	Yes	166 (28.6)	39 (26.9)	39 (26.9)	42 (28.8)	46 (31.7)	
Drink, *n* (%)	No	422 (72.6)	98 (67.1)	103 (71.0)	108 (74.5)	113 (77.9)	0.194
	Yes	159 (27.4)	48 (32.9)	42 (29.0)	37 (25.5)	32 (22.1)	
Smoke, *n* (%)	No	329 (56.6)	81 (55.5)	76 (52.4)	80 (55.2)	92 (63.4)	0.262
	Yes	252 (43.4)	65 (44.5)	69 (47.6)	65 (44.8)	53 (36.6)	
ALB, g/L	–	42.7 (38.30, 45.40)	42.0 (38.20, 44.9)	43.50 (39.80, 46.10)	42.1 (37.2, 44.9)	42.8 (38.1, 45.1)	0.179
CRP, mg/L	–	29.0 (5.4, 85.8)	12.4 (3.47, 63.52)	17.40 (4.00, 68.00)	47.20 (7.50, 133.30)	43.50 (7.25, 81.40)	0.001
TC, mmol/L	–	5.51 (4.3, 7.4)	5.22 (4.19, 6.36)	5.31 (4.34, 6.99)	5.87 (4.41, 7.95)	5.6 (4.16, 7.85)	0.07
LDL, mmol/L	–	2.9 (2.21, 3.53)	2.90 (2.49, 3.59)	2.9 (2.28, 3.62)	2.9 (2.26, 3.51)	2.66 (1.56, 3.23)	0.003
TG, mmol/L	–	3.31 (1.17, 15.53)	1.96 (1.09, 6.96)	3.98 (1.35, 15.56)	4.21 (1.31, 17.54)	4.83 (1.03, 18.81)	0.014
WBC, 10^9^/L	–	11.0 (8.40, 13.7)	7.4 (5.73, 9.9)	11.0 (8.8, 12.5)	12.2 (10.0, 14.1)	14.1 (11.10, 17.8)	<0.001
PLT, 10^9^/L	–	229.0 (187.0, 278.0)	227.0 (190.0, 283.5)	241.0 (198.0, 286.0)	221.0 (180.0, 267.0)	222.0 (173.0, 268.0)	0.051
HCT, %	–	42.0 (38.0, 45.0)	41.0 (37.0, 44.0)	42.0 (39.0, 45.0)	42.0 (38.00, 45.0)	43.0 (39.0, 46.0)	0.005
LYM, 10^9^/L	–	1.3 (0.8, 1.8)	1.9 (1.4, 2.4)	1.6 (1.3, 2.0)	1.1 (0.9, 1.4)	0.7 (0.5, 0.8)	<0.001
MONO, 10^9^/L	–	0.50 (0.4, 0.8)	0.50 (0.4, 0.6)	0.60 (0.4, 0.7)	0.60 (0.4, 0.8)	0.6 (0.4, 0.9)	<0.001
Neut, 10^9^/L	–	8.70 (6.10, 11.60)	4.50 (3.22, 6.4)	8.60 (6.8, 9.9)	10.00 (8.5, 11.9)	12.5 (9.3, 15.8)	<0.001
RDW	–	40.5 (38.4, 42.2)	40.5 (38.92, 42.88)	40.5 (38.3, 42.0)	40.50 (38.6, 42.1)	40.1 (38.1, 42.0)	0.406
D-D, mg/L	–	0.67 (0.28, 1.74)	0.54 (0.21, 1.45)	0.52 (0.26, 1.50)	0.77 (0.32, 1.75)	0.85 (0.37, 2.14)	0.004
Ascites, *n* (%)	No	391 (67.3)	117 (80.1)	108 (74.5)	82 (56.6)	84 (57.9)	<0.001
	Yes	190 (32.7)	29 (19.9)	37 (25.5)	63 (43.4)	61 (42.1)	
Gallstones, *n* (%)	No	405 (69.7)	111 (76.0)	112 (77.2)	96 (66.2)	86 (59.3)	0.002
	Yes	176 (30.3)	35 (24.0)	33 (22.8)	49 (33.8)	59 (40.7)	

### 3.2 Association between NLR and liver injury

**FIGURE 1:**
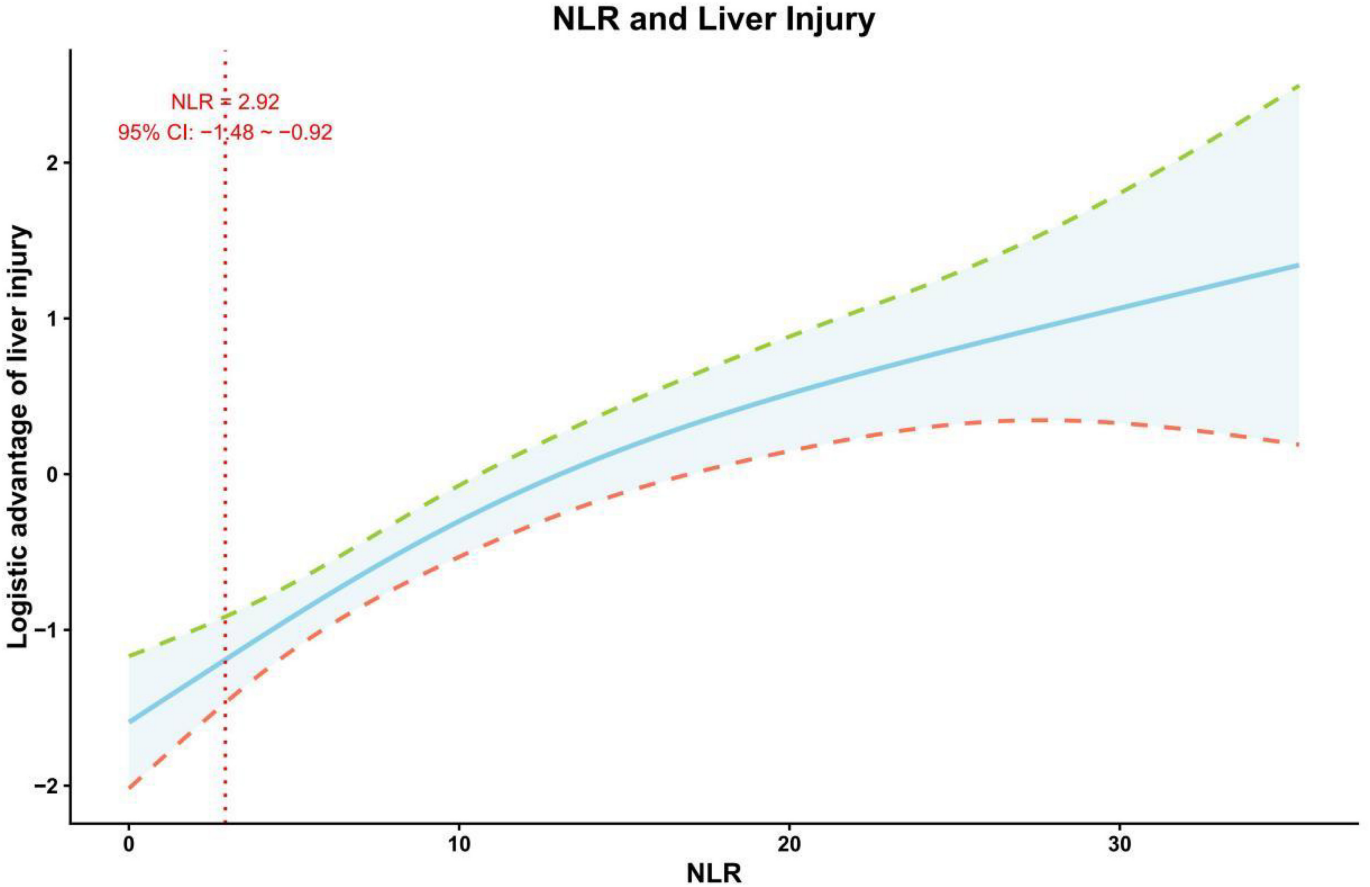
Smoothing curve of the association between neutrophil-to-lymphocyte ratio (NLR) and liver injury in acute pancreatitis patients (generalized additive model).

A multivariable logistic regression analysis was conducted to examine the association between NLR and ALI in patients with AP, using the lowest NLR quartile as the reference group. Before adjusting for confounding factors, the odds ratios (ORs) with 95% confidence intervals (CIs) for ALI risk in the second, third, and fourth quartiles were 1.93 (1.14–3.33), 2.93 (1.75–5.00), and 5.25 (3.14–8.96), respectively. After adjusting for age alone, the corresponding ORs (95% CI) were 1.93 (1.13–3.32), 2.92 (1.74–4.98), and 5.37 (3.21–9.19). After further adjustment for smoking history, alcohol consumption, gallstones, diabetes, hyperlipidemia, and ascites, the associations remained significant across all quartiles, with ORs (95% CI) of 2.11 (1.18–3.80), 2.88 (1.62–5.21), and 5.42 (3.05–9.86), respectively. When NLR was treated as a continuous variable in the multivariable logistic regression model and adjusted for the same confounders as in Model 3, each one-unit increase in NLR was associated with an 11% increase in the risk of liver injury (OR = 1.11, 95% CI: 1.08–1.14, *P* < 0.001). A generalized additive model with smoothing curve fitting was used to analyze the non-linear association between NLR and ALI, and the results demonstrated a significant nonlinear relationship between the two variables (Figure 1).

**FIGURE 2:**
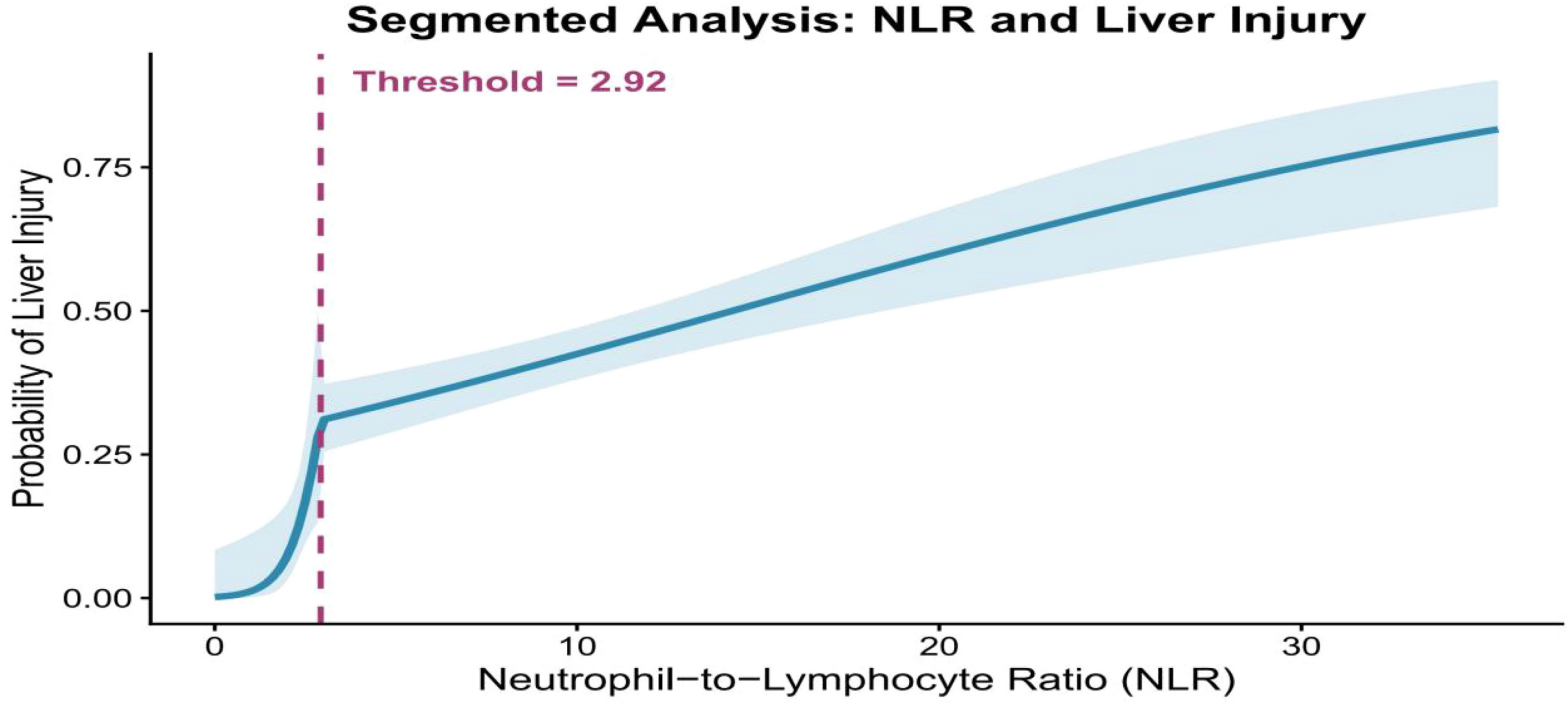
Neutrophil-to-lymphocyte ratio (NLR) and liver injury: segmented regression.

Threshold effect analysis revealed a significant non-linear threshold relationship between NLR and the risk of liver injury. The linear logistic regression model showed that each one-unit increase in NLR was associated with a significantly increased risk of liver injury (OR = 1.11, 95% CI: 1.08–1.14, *P* < 0.001). The two-piecewise logistic regression model identified an inflection point for NLR at 2.92. When NLR was below 2.92, the risk of liver injury increased sharply with rising NLR (OR = 6.55, 95% CI: 1.33–32.20, *P* = 0.021); when NLR was ≥2.92, the increasing trend in risk slowed significantly (OR = 1.07, 95% CI: 1.04–1.10, *P* < 0.001). The likelihood ratio test confirmed that the two-piecewise non-linear model provided a significantly better fit than the linear model (χ^2^ = 20.484, df = 2, *P* < 0.001), indicating that the association between NLR and liver injury is non-linear and exhibits a clear threshold effect (Figure 2 and Table 3).

**TABLE 3:** Threshold effect analysis of neutrophil-to-lymphocyte ratio (NLR) in relation to liver injury.

Liver injury	Adjusted OR (95% CI)	*P*-value
NLR	–	–
Fitting by the standard linear model	1.11 (1.08–1.14)	<0.001
Fitting by a two-piecewise linear model	–	–
Inflection point	2.92	–
<2.92	6.55 (1.33–32.20)	0.021
≥2.92	1.07 (1.04–1.10)	<0.001
Log-likelihood ratio	–	<0.001

### 3.3 Subgroup analysis

**FIGURE 3:**
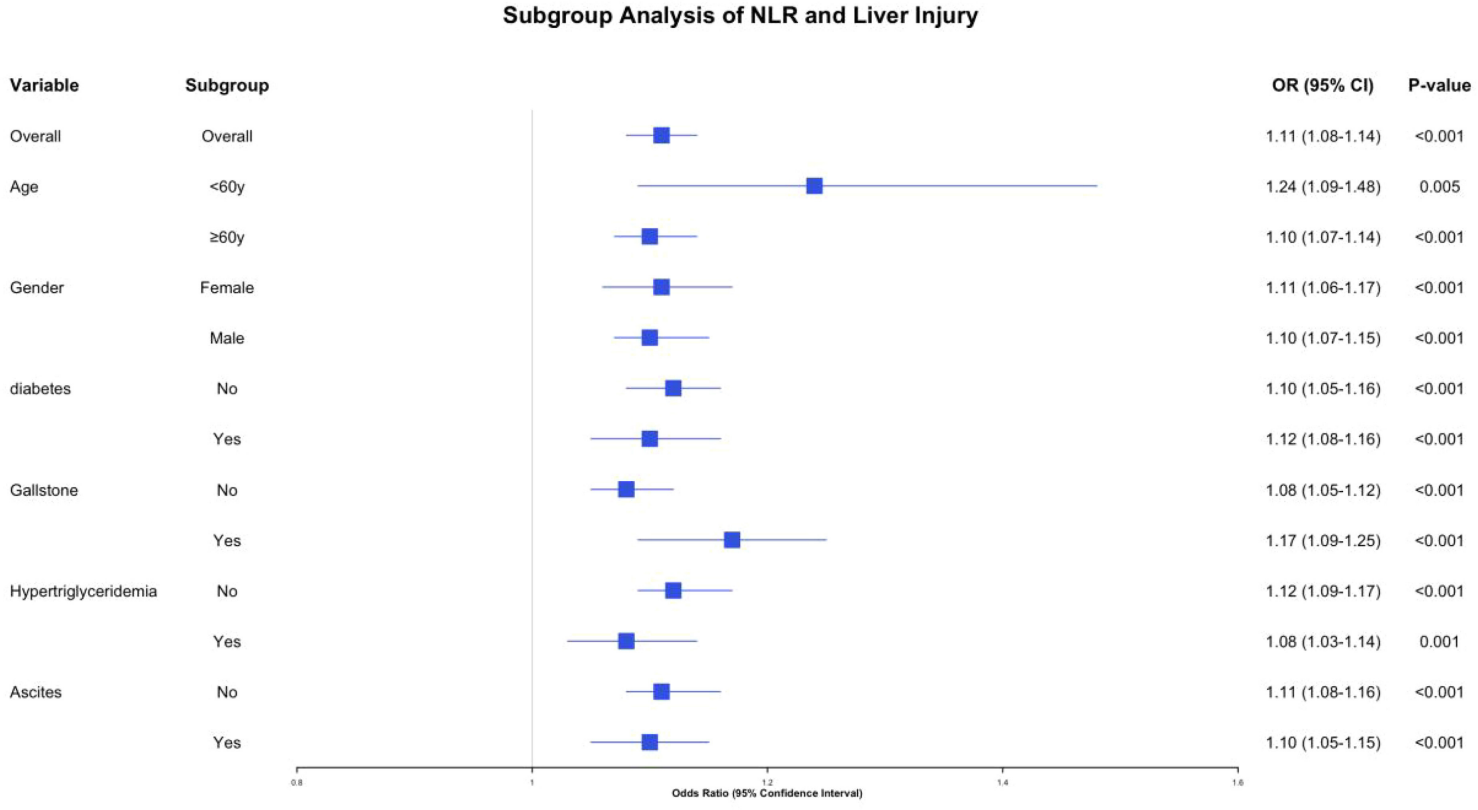
Subgroup analysis of the association between neutrophil-to-lymphocyte ratio (NLR) and liver injury.

Subgroup analyses and interaction tests were performed for age, sex, diabetes, gallstones, hyperlipidemia, and ascites, with adjustments for age, sex, alcohol consumption history, smoking history, diabetes, hyperlipidemia, and ascites. In the overall population, after adjusting for confounding factors, each one-unit increase in NLR was associated with an 11% increase in the risk of liver injury (OR = 1.11, 95% CI: 1.08–1.14, *P* < 0.001). In the age < 60 years subgroup, the OR was 1.24 (95% CI: 1.09–1.48); in the age ≥ 60 years subgroup, the OR was 1.10 (95% CI: 1.07–1.14). In the male subgroup, the OR was 1.10 (95% CI: 1.07–1.15); in the female subgroup, the OR was 1.11 (95% CI: 1.06–1.17). In patients with diabetes, the OR was 1.10 (95% CI: 1.05–1.16); in those without diabetes, the OR was 1.12 (95% CI: 1.08–1.16). In the hyperlipidemia subgroup, the OR was 1.08 (95% CI: 1.03–1.14); in the non-hyperlipidemia subgroup, the OR was 1.12 (95% CI: 1.09–1.17). In patients with gallstones, the OR was 1.17 (95% CI: 1.09–1.25); in those without gallstones, the OR was 1.08 (95% CI: 1.05–1.12). In the ascites subgroup, the OR was 1.10 (95% CI: 1.05–1.15); in the non-ascites subgroup, the OR was 1.11 (95% CI: 1.08–1.16). All subgroup associations were statistically significant (all *P* < 0.01) (Figure 3).

Results of the interaction analysis showed that there were no statistically significant differences in the association between NLR and liver injury across the subgroups (all *P* > 0.05), indicating that the aforementioned factors did not significantly modify this association. Specifically, age (*P* = 0.086), sex (*P* = 0.706), diabetes (*P* = 0.497), gallstones (*P* = 0.069), hyperlipidemia (*P* = 0.155), and ascites (*P* = 0.883) all lacked statistical significance, suggesting that the association between NLR and liver injury remained consistent across all subgroups.

### 3.4 Receiver operating characteristic curve

**FIGURE 4:**
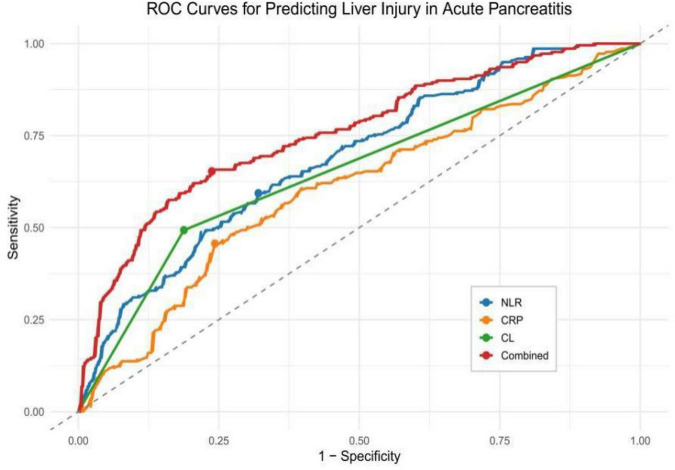
Receiver operating characteristic (ROC) curves for predicting liver injury in acute pancreatitis.

Receiver operating characteristic curve analysis showed that the AUC of NLR for predicting liver injury in patients with AP was 0.688 (95% CI: 0.644–0.731), with an optimal cutoff value of 8.26, corresponding to a sensitivity of 59.4% and a specificity of 68.0%. The AUCs for CRP and the presence of gallstones as standalone predictors were 0.605 (95% CI: 0.557–0.653) and 0.653 (95% CI: 0.614–0.691), respectively. The AUC of the combined predictive probability model increased to 0.756 (95% CI: 0.715–0.797), with a sensitivity of 65.8% and a specificity of 76.2% (Figure 4 and Table 4).

**TABLE 4:** Receiver operating characteristic (ROC) analysis results for predicting liver injury in acute pancreatitis.

Indicator	AUC (95% CI)	Cut-off	Sensitivity (%)	Specificity (%)
NLR	0.688 (0.644–0.731)	8.26	59.4	68.0
CRP	0.605 (0.557–0.653)	66.05 mg/L	45.7	75.7
CL (cholelithiasis)	0.653 (0.614–0.691)	–	49.3	81.2
Combined	0.756 (0.715–0.797)	–	65.8	76.2

## 4 Discussion

This retrospective study analyzed the association between NLR and the risk of liver injury in patients with AP. The results revealed that an elevated NLR was independently associated with an increased risk of liver injury in patients with AP, and that this relationship was non-linear. Specifically, when NLR was below 2.92, the risk of liver injury increased sharply with rising NLR. No significant interactions were observed between NLR and any of the subgroup variables. ROC curve analysis evaluated the predictive performance of NLR both as a standalone marker and in combination with other indicators for liver injury in patients with AP. In summary, NLR may serve as a potential screening marker for early risk identification of liver injury in patients with AP.

Pancreatitis-associated liver injury results from the interaction of multiple factors. Pancreatic enzymes can directly damage hepatocytes via the portal vein (20); the inflammatory cascade activates Kupffer cells, leading to the release of cytokines such as TNF-α and IL-6, which induce oxidative stress and lipid peroxidation (21–23). Concurrently, microcirculatory disturbances leading to ischemia-reperfusion injury, compression of the common bile duct causing bile stasis, and disruption of the intestinal barrier resulting in endotoxin translocation can all exacerbate liver damage (24, 25). Building on this, Yang et al. (26) were the first to clearly identify pancreatitis-associated ascites as an independent key mediator of liver injury and fully elucidated the apoptotic pathway: mitochondrial damage → p38-MAPK → caspase-3. Rao et al. (27) confirmed that the presence of ascites is a significant risk factor for liver injury in patients with acute pancreatitis (AP), and that the characteristics of ascites are significantly correlated with the severity of liver injury. The study by Li et al. (28) further demonstrated that the occurrence of severe acute pancreatitis (SAP)-associated liver injury is closely related to intensified systemic inflammatory responses, increased oxidative stress, and activation of the NLRP3 inflammasome. These mechanisms indicate that the level of systemic inflammation is a key determinant of the severity of liver injury, thereby explaining why NLR, as an inflammatory marker, can predict the risk of liver injury in AP patients.

Similar to other critical illnesses, the inflammatory response triggered by AP follows a distinct progression pattern. Previous studies have indicated that inflammation typically progresses rapidly within the first week of onset (29); this phase represents a critical window for preventing the worsening of liver injury and improving prognosis, necessitating the use of inflammatory markers for early risk assessment in clinical practice. Within this complex pathophysiological process, the intensity of the systemic inflammatory response is one of the key determinants of whether liver injury occurs. As a simple indicator reflecting systemic inflammation levels, NLR holds promise for assessing the risk of liver injury.

The NLR reflects systemic inflammatory responses and immune imbalance in the body and serves as a reliable surrogate marker for predicting the risk of liver injury. The results of this study show that elevated NLR levels in patients with AP are significantly associated with an increased prevalence of liver injury. After adjusting for confounding factors, the risk of ALI in patients in the highest NLR quartile was 5.42 times higher than that in the lowest quartile. Previous studies further support the conclusions of this study from both mechanistic and clinical application perspectives. Studies have confirmed that NLR measured on days 2 and 3 of AP onset is significantly positively correlated with multiple inflammatory markers; this indicator can replace more expensive tests for the early assessment of organ failure risk (30). Persistently elevated NLR within 48 h of admission has been confirmed as an independent risk factor for the progression of AP to severe disease. Owing to its ease of testing and good negative predictive value, NLR is suitable for early rapid patient stratification (31). Multiple studies consistently indicate that NLR can independently assess the severity of AP, and when combined with other indicators, it can further enhance overall predictive performance (32). Zhang et al. (33) demonstrated that an elevated admission NLR independently predicted persistent organ failure, prolonged ICU stay and in-hospital mortality in a cohort of 974 Asian Chinese patients with acute pancreatitis. NLR measurements at different time points each have distinct clinical advantages: the 24-h NLR offers the best predictive performance for severe AP, while the 48 h NLR is particularly valuable for predicting mortality and the risk of organ failure (34). In summary, NLR not only serves as an independent predictor of AP complicated by liver injury but also effectively assesses disease severity, complications, and mortality risk. This biomarker is simple to measure and provides stable results; when combined with other biological markers, it can further optimize clinical risk stratification and early warning systems for patients with AP.

This study confirms a non-linear threshold effect between NLR and the risk of ALI in patients with AP, with an inflection point of 2.92. Previous studies have primarily focused on the predictive value of NLR for the risk of severe AP and mortality, and the threshold values reported in those studies have generally been higher. Halaseh et al. (35), based on data from 314 patients with AP, found that an admission NLR > 18.71 had a sensitivity of 80% and a specificity of 90.2% for predicting mortality; simultaneously, a persistently elevated NLR within 24–48 h was significantly associated with the risk of complications. Salehi et al. (36) used an NLR of 6.23 as the cutoff value for predicting moderate-to-severe AP. Kumar et al. (37) reported that an NLR > 5.66 yielded an AUC of 0.998 for predicting severe AP. Aydın et al. (38) also confirmed that NLR levels are significantly elevated in patients with AP. Another study showed that an admission NLR > 7.13 had an AUC of 0.81 for predicting systemic complications in AP (39). Unlike the aforementioned reports, which used severe disease, death, or systemic complications as study endpoints, the present study used ALI as the outcome measure and identified a lower NLR inflection point. These results suggest that the risk of liver injury in patients with AP begins to rise significantly even when the systemic inflammatory response is still in its early stages. This conclusion further broadens the clinical application of NLR: for patients with AP who have an admission NLR ≥ 2.92, even in the absence of current signs of severe illness, enhanced dynamic monitoring of liver function and timely early intervention are warranted to prevent or delay the onset and progression of ALI.

This study conducted subgroup analyses for age, sex, diabetes, gallstones, hypertriglyceridemia, and ascites. The results showed that the association between NLR and liver injury remained stable across all subgroups, with no significant interactions. This indicates that the predictive value of NLR is not significantly influenced by factors such as age, underlying diseases, or complications. Therefore, NLR remains applicable in complex clinical scenarios involving elderly patients, patients with diabetes or gallstones, or those with ascites, demonstrating good generalizability and clinical utility.

The AUC for NLR alone in predicting ALI in AP patients was 0.688, indicating moderate predictive value. However, after combining NLR with CRP and gallstones, the AUC increased to 0.756, showing that combining common clinical indicators significantly improves predictive performance. This finding is supported by prior studies. Karabuga et al. (40) confirmed that NLR combined with other inflammatory markers enhances predictive ability. Xu et al. (41) found that NLR plus CRP yielded an AUC of 0.873, and adding TyG further increased the AUC to 0.882. A large study reported that NLR predicted persistent organ failure in AP with AUCs of 0.619–0.668 (42), consistent with our result (0.688), supporting NLR as a screening tool. Other cohort studies have also validated NLR’s predictive performance: Park et al. (43) reported an AUC of 0.821 for severe AP, while Jeon and Park (44) found AUCs of 0.59 and 0.62 for SAP and organ failure, respectively, and noted that persistently elevated NLR indicates poor prognosis. Dancu et al. (45) showed that combining 48h NLR with 48h CRP improves diagnostic accuracy. Maghsoomi et al. (46) reported that dynamic NLR monitoring identifies mortality risk early (AUC 0.87). Systematic reviews corroborate these findings: Chooklin and Chuklin (47) concluded that NLR has moderate discriminatory performance, with dynamic monitoring further optimizing its value; Vo et al. (48) noted that NLR alone is not inferior to some combined indicators.

Neutrophil-to-lymphocyte ratio also has value in specific pancreatitis types: high NLR is an independent risk factor for progression in hypertriglyceridemia-induced AP (49). Combined testing offers advantages: NLR plus CRP predicts organ failure with an AUC of 0.65 (cutoff 8.41) and triples the risk of organ failure (50); NLR plus CRP predicts SAP with an AUC of 0.866, providing a simple method for early identification of multiple organ failure (51). Gallstones, the most common cause of AP, can induce systemic inflammation and increase ALI risk (52); combining gallstone history with NLR enhances assessment of AP-related adverse events (53). In summary, NLR and CRP reflect inflammatory burden. Combining etiological factors with inflammatory markers enables multidimensional assessment, explaining why combined models outperform single indicators. NLR shows consistent screening value for AP-associated liver injury and overall severity. In clinical practice, NLR should be combined with easily available indicators such as CRP and gallstone history to improve early identification of adverse events.

It is important to note that the threshold effect inflection point (2.92) and the optimal ROC cutoff value (8.26) serve different clinical purposes. The former describes the turning point in the relationship between NLR and ALI risk, used to identify the population in whom the risk begins to rise rapidly; the latter is the cutoff value obtained by maximizing the Youden index in a screening context, used to distinguish between high- and low-risk patients for ALI. The two are not contradictory but rather complementary: clinically, an NLR ≥ 2.92 can first serve as an early warning signal, followed by an NLR ≥ 8.26 as a reference for further risk stratification.

## 5 Limitations and future directions

Given the current research conditions and advancements in similar international studies, this study still has several limitations. (1) The study employed a single-center retrospective design, which cannot establish a definitive causal relationship between NLR and liver injury in acute pancreatitis; furthermore, selection bias is present, limiting the generalizability of the conclusions. (2) Only a single NLR measurement at admission was recorded, and no dynamic monitoring was conducted, which may underestimate its predictive value over time. (3) Although major confounding factors were adjusted for, residual confounding remains, as factors such as alcohol intake, nutritional status, and pre-admission medication use (e.g., anti-inflammatory agents, hepatotoxic drugs) were not included in the analysis. (4) The predictive performance of NLR alone is moderate, and there is room for optimization of the combined model. (5) No stratification analysis was performed based on etiology, and the predictive differences of this indicator across different subgroups remain unclear.

In line with international research trends, future studies could be deepened in several areas: (1) Conducting multicenter prospective studies to dynamically monitor changes in NLR at different time points and analyze its association with liver injury. (2) Combining multiple laboratory indicators and clinical scores to optimize composite predictive models. (3) Supplementing with etiology-based stratification analysis to clarify the indicator’s application value across different subtypes. (4) Utilizing cutting-edge modeling methods such as machine learning to further explore the data value of the indicator and improve the accuracy of predictive models. (5) Preliminarily exploring the mechanisms underlying NLR’s involvement in liver injury, and elucidating the specific pathways through which NLR contributes to the onset and progression of liver injury in acute pancreatitis from a pathophysiological perspective.

## 6 Conclusion

Neutrophil-to-lymphocyte ratio is an independent predictor of liver injury in patients with acute pancreatitis, and a clear non-linear threshold effect exists between the two. When NLR is mildly elevated, the risk of liver injury increases rapidly; after exceeding the inflection point, the upward trend in risk slows, suggesting that early inflammatory responses have a significant impact on liver function. It is recommended that an admission NLR ≥ 2.92 be used as an early warning signal to initiate dynamic monitoring of liver function, and that an NLR ≥ 8.26 serve as a reference for stratifying high-risk patients. The predictive performance of NLR alone for liver injury is moderate; however, its predictive ability is significantly enhanced when combined with CRP and the presence of gallstones, resulting in both sensitivity and specificity superior to those of a single indicator. Therefore, in clinical practice, NLR should be used in combination with other readily available inflammatory markers to optimize early screening and stratified management of liver injury in acute pancreatitis, thereby improving patient outcomes.

## Data Availability

The original contributions presented in this study are included in this article/supplementary material, further inquiries can be directed to the corresponding authors.

## References

[1] Lai T Li J Zhou Z Rao J Zhu Y Xia L et al. Etiological changes and prognosis of hospitalized patients with acute pancreatitis over a 15-year period. *Dig Dis Sci*. (2024) 69:56–65. 10.1007/s10620-023-08172-0 37943383

[2] Sohail Z Shaikh H Iqbal N Parkash O. Acute pancreatitis: a narrative review. *J Pak Med Assoc*. (2024) 74:953–8. 10.47391/JPMA.9280 38783446

[3] Liu W Du JJ Li ZH Zhang XY Zuo HD. Liver injury associated with acute pancreatitis: the current status of clinical evaluation and involved mechanisms. *World J Clin Cases*. (2021) 9:10418–29. 10.12998/wjcc.v9.i34.10418 35004974 PMC8686151

[4] Hu WM Hua TR Zhang YL Chen GR Song K Pendharkar S et al. Prognostic significance of organ failure and infected pancreatic necrosis in acute pancreatitis: an updated systematic review and meta-analysis. *J Dig Dis*. (2023) 24:648–59. 10.1111/1751-2980.13243 38037512

[5] Yu L Xie F Luo L Lei Y Huang X Yang X et al. Clinical characteristics and risk factors of organ failure and death in necrotizing pancreatitis. *BMC Gastroenterol*. (2023) 23:19. 10.1186/s12876-023-02651-4 36658497 PMC9850524

[6] Hoferica J Borbély RZ Aghdam AN Szalai EÁ Zolcsák Á Veres DS et al. Chronic liver disease is an important risk factor for worse outcomes in acute pancreatitis: a systematic review and meta-analysis. *Sci Rep*. (2024) 14:16723. 10.1038/s41598-024-66710-w 39030187 PMC11271551

[7] Peng Q Long Y Yang K Yang K Jia J Hu S et al. Prognostic analysis of acute pancreatitis due to different etiologies and establishment and validation of an early prediction model. *Lipids Health Dis.* (2026) 25:74. 10.1186/s12944-026-02890-w 41645226 PMC12964949

[8] Yu M Zhang N Chen C He Y Meng J Luo W et al. Inflammatory ratios as early predictors of disease severity and immunotherapy response in autoimmune encephalitis. *J Neuroimmunol*. (2026) 410:578786. 10.1016/j.jneuroim.2025.578786 41161144

[9] Carollo C Sorce A Cirafici E Ciuppa ME Mulè G Caimi G. Silent inflammation, loud consequences: decoding NLR across renal, cardiovascular and metabolic disorders. *Int J Mol Sci*. (2025) 26:8256. 10.3390/ijms26178256 40943179 PMC12428752

[10] Chen X Yang X Zhang M Zhao Y Guo S. Neutrophil-lymphocyte and platelet-lymphocyte ratios as systemic inflammatory biomarkers for atopic dermatitis in US adults: a cross-sectional NHANES study revealing subgroup heterogeneity. *Front Immunol*. (2025) 16:1585451. 10.3389/fimmu.2025.1585451 40607418 PMC12213362

[11] Lin Z Chen Y Long J Zhang X. Elevated Serum DcR3, Fas, and NLR Ratio are correlated with the progression of Liver Cirrhosis. *Am J Transl Res*. (2025) 17:1057–64. 10.62347/HWYM7712 40092078 PMC11909560

[12] Jing C Zhao Z Xin Y Long J. The predictive value of NLR and PLR for TACE prognosis in hepatocellular carcinoma: a systematic review and meta-analysis. *Open Med (Wars)*. (2026) 21:20261378. 10.1515/med-2026-1378 42109905 PMC13150366

[13] Felismino T Martins L Barroso M Carvalho D Brito A Maccali C et al. Neutrophil-to-Lymphocyte Ratio (NLR) as a predictive biomarker in advanced hepatocellular carcinoma treated with first-line immunotherapy. *J Gastrointest Cancer*. (2025) 56:175. 10.1007/s12029-025-01299-5 40830281

[14] Zahorec R. Neutrophil-to-lymphocyte ratio, past, present and future perspectives. *Bratisl Lek Listy.* (2021) 122:474–88. 10.4149/BLL_2021_078 34161115

[15] Pian G Li H Piao Y. Clinical significance of inflammation markers in predicting the severity of acute pancreatitis. *Pancreas*. (2021) 50:201–5. 10.1097/MPA.0000000000001749 33565796

[16] Cho SK Jung S Lee KJ Kim JW. Neutrophil to lymphocyte ratio and platelet to lymphocyte ratio can predict the severity of gallstone pancreatitis. *BMC Gastroenterol*. (2018) 18:18. 10.1186/s12876-018-0748-4 29370777 PMC5785858

[17] Iap/Apa/Epc/Ipc/Jps Working Group. International association of pancreatology revised guidelines on acute pancreatitis 2025: supported and endorsed by the American Pancreatic Association, European Pancreatic Club, Indian Pancreas Club, and Japan Pancreas Society. *Pancreatology.* (2025) 25:770–814. 10.1016/j.pan.2025.04.020 40651900

[18] Li R Tang Y Liang M Ding J. Liver injury in COVID-19 patients with metabolic syndrome-a narrative review. *Ann Palliat Med*. (2021) 10:8264–70. 10.21037/apm-21-1398 34353107

[19] Zahorec R. Ratio of neutrophil to lymphocyte counts–rapid and simple parameter of systemic inflammation and stress in critically ill. *Bratisl Lek Listy.* (2001) 102:5–14. 11723675

[20] Hirano T Manabe T Tobe T. Impaired hepatic energy metabolism in rat acute pancreatitis: protective effects of prostaglandin E2 and synthetic protease inhibitor ONO 3307. *J Surg Res*. (1992) 53:238–44. 10.1016/0022-4804(92)90041-w 1528049

[21] Feng Y Jin X Xu H Sun B Guo M Li M. Pancreatic elastase affects liver injury by activating pro-inflammatory cytokines in Kupffer cells via the JAK2/STAT3 signaling pathway. *Curr Mol Med.* (2025). Published online July 1, 2025. 10.2174/0115665240363959250624052201 40605170 PMC13478933

[22] Ueda T Takeyama Y Takase K Hori Y Kuroda Y Ho HS. Hematin is one of the cytotoxic factors in pancreatitis-associated ascitic fluid that causes hepatocellular injury. *Surgery*. (2002) 131:66–74. 10.1067/msy.2002.118317 11812965

[23] Zhao H Luo Y Chen X Wu J Zhu Z Chen H. TAGLN2 exacerbates acute pancreatitis-induced liver injury by increasing hepatocyte pyroptosis via Kupffer cells-mediated inflammatory response. *Arch Immunol Ther Exp (Warsz).* (2025) 73:10.2478/aite–2025–0014. 10.2478/aite-2025-0014 40472315

[24] Oliver L Liu C Sadowski BA. Case of recurrent liver injury-associated acute pancreatitis (LIAAP). *Cureus.* (2024) 16:e65272. 10.7759/cureus.65272 39184768 PMC11343480

[25] Liu X Zheng Y Meng Z Wang H Zhang Y Xue D. Gene regulation of neutrophils mediated liver and lung injury through NETosis in acute pancreatitis. *Inflammation*. (2025) 48:393–411. 10.1007/s10753-024-02071-w 38884700

[26] Yang J Fier A Carter Y Liu G Epling-Burnette PK Bai F et al. Liver injury during acute pancreatitis: the role of pancreatitis-associated ascitic fluid (PAAF), p38-MAPK, and caspase-3 in inducing hepatocyte apoptosis. *J Gastrointest Surg*. (2003) 7:200–7. 10.1016/s1091-255x(02)00134-8 12600444

[27] Rao JW Li JR Wu Y Lai TM Zhou ZG Gong Y et al. Ascites characteristics in acute pancreatitis: a prognostic indicator of organ failure and mortality. *World J Gastroenterol*. (2025) 31:108926. 10.3748/wjg.v31.i28.108926 40741476 PMC12305166

[28] Li Z Yu Y Zhao X Qu Y Wang J Zhang D. Chaperone-mediated autophagy reactivation protects against severe acute pancreatitis-associated liver injury through upregulating Keap1/Nrf2 signaling pathway and inhibiting NLRP3 inflammasome activation. *Cell Biochem Biophys*. (2025) 83:2919–35. 10.1007/s12013-025-01677-7 39998716

[29] Choudhury A Kumar M Sharma BC Maiwall R Pamecha V Moreau R et al. Systemic inflammatory response syndrome in acute-on-chronic liver failure: relevance of ‘golden window’: a prospective study. *J Gastroenterol Hepatol*. (2017) 32:1989–97. 10.1111/jgh.13799 28374414

[30] Kolber W Kuśnierz-Cabala B Maraj M Kielar M Mazur P Maziarz B et al. Neutrophil to lymphocyte ratio at the early phase of acute pancreatitis correlates with serum urokinase-type plasminogen activator receptor and interleukin 6 and predicts organ failure. *Folia Med Cracov.* (2018) 58:57–74. 30745602

[31] Suppiah A Malde D Arab T Hamed M Allgar V Smith AM et al. The prognostic value of the neutrophil-lymphocyte ratio (NLR) in acute pancreatitis: identification of an optimal NLR. *J Gastrointest Surg.* (2013) 17:675–81. 10.1007/s11605-012-2121-1 23371356

[32] Zhou H Li W Yang S Yang H Cai Y. Prediction of acute pancreatitis severity using NLR, procalcitonin, and CT severity score: a retrospective study. *Medicine (Baltimore)*. (2025) 104:e43055. 10.1097/MD.0000000000043055 40629608 PMC12237365

[33] Zhang Y Wu W Dong L Yang C Fan P Wu H. Neutrophil to lymphocyte ratio predicts persistent organ failure and in-hospital mortality in an Asian Chinese population of acute pancreatitis. *Medicine (Baltimore)*. (2016) 95:e4746. 10.1097/MD.0000000000004746 27631223 PMC5402566

[34] Bengi G Çelik İ Dolu S Önem S Soytürk M Rendeci S et al. Neutrophil-lymphocyte ratio and LDH/albumin ratio as biomarkers for severity and mortality in acute pancreatitis. *Turk J Gastroenterol*. (2025) 36:497–507. 10.5152/tjg.2025.24828 40521831 PMC12351294

[35] Halaseh SA Kostalas M Kopec C Toubasi AA Salem R. Neutrophil-to-lymphocyte ratio as an early predictor of complication and mortality outcomes in individuals with acute pancreatitis at a UK district general hospital: a retrospective analysis. *Cureus.* (2022) 14:e29782. 10.7759/cureus.29782 36340538 PMC9621742

[36] Salehi AM Rezaei R Sadeghi A Ayubi E Hasanzarrini M Azandaryani AR et al. Diagnostic value of neutrophil/lymphocyte ratio, platelet/lymphocyte ratio and systemic immune-inflammation index for predicting the severity of acute pancreatitis. *BMC Gastroenterol.* (2025) 25:751. 10.1186/s12876-025-04370-4 41126061 PMC12542137

[37] Kumar S Ekka NMP Sinha DK Kumar B Kujur ADS Mayank S et al. Neutrophil/Lymphocyte ratio an accurate and simple tool to identify severe acute pancreatitis and predict mortality risk. *J Family Med Prim Care*. (2025) 14:4574–9. 10.4103/jfmpc.jfmpc_1959_24 41403525 PMC12704992

[38] Aydin G Akdogan RA Rakici H Akdogan E. Predictive value of immature granulocyte percentage and neutrophil lymphocyte ratio in terms of prognosis in the course of acute pancreatitis. *Bratisl Lek Listy*. (2024) 125:477–83. 10.4149/BLL_2024_74 38989748

[39] Kokulu K Günaydın YK AkYK NB Köylü R Sert ET Köylü Ö et al. Relationship between the neutrophil-to-lymphocyte ratio in acute pancreatitis and the severity and systemic complications of the disease. *Turk J Gastroenterol.* (2018) 29:684–91. 10.5152/tjg.2018.17563 30381275 PMC6284671

[40] Karabuga B Gemcioglu E Konca Karabuga E Baser S Ersoy O. Comparison of the predictive values of CRP, CRP/albumin, RDW, neutrophil/lymphocyte, and platelet/lymphocyte levels in determining the severity of acute pancreatitis in patients with acute pancreatitis according to the BISAP score. *Bratisl Lek Listy*. (2022) 123:129–35. 10.4149/BLL_2022_020 35065589

[41] Xinyu X Jiang Z Qing A Lihua L Xiehong L Lin Z. Clinical significance of PCT, CRP, IL-6, NLR, and TyG Index in early diagnosis and severity assessment of acute pancreatitis: a retrospective analysis. *Sci Rep*. (2025) 15:2924. 10.1038/s41598-025-86664-x 39849025 PMC11758003

[42] Xu MS Xu JL Gao X Mo SJ Xing JY Liu JH et al. Clinical study of neutrophil-to-lymphocyte ratio and platelet-to-lymphocyte ratio in hypertriglyceridemia-induced acute pancreatitis and acute biliary pancreatitis with persistent organ failure. *World J Gastrointest Surg*. (2024) 16:1647–59. 10.4240/wjgs.v16.i6.1647 38983313 PMC11230014

[43] Park HS In SG Yoon HJ Lee WJ Woo SH Kim D. Predictive values of neutrophil-lymphocyte ratio as an early indicator for severe acute pancreatitis in the emergency department patients. *J Lab Physicians.* (2019) 11:259–64. 10.4103/JLP.JLP_82_19 31579249 PMC6771314

[44] Jeon TJ Park JY. Clinical significance of the neutrophil-lymphocyte ratio as an early predictive marker for adverse outcomes in patients with acute pancreatitis. *World J Gastroenterol*. (2017) 23:3883–9. 10.3748/wjg.v23.i21.3883 28638228 PMC5467074

[45] Dancu GM Popescu A Sirli R Danila M Bende F Tarta C et al. The BISAP score, NLR, CRP, or BUN: which marker best predicts the outcome of acute pancreatitis? *Medicine (Baltimore)*. (2021) 100:e28121. 10.1097/MD.0000000000028121 34941057 PMC8702250

[46] Maghsoomi Z Shahbeigy S Tajik Rostami F. NLR and platelet dynamics: simple, cost-effective biomarkers for stratifying severity in acute pancreatitis. *Med J Islam Repub Iran*. (2025) 39:152. 10.47176/mjiri.39.152 42021808 PMC13097221

[47] Chooklin S Chuklin S. Neutrophil-to-lymphocyte ratio for primary risk stratification in acute pancreatitis: a systematic review and meta-analysis. *Front Med (Lausanne).* (2026) 12:1729339. 10.3389/fmed.2025.1729339 41608433 PMC12835348

[48] Vo HH Truong-Thi NN Ho-Thi HB Vo HMC Tran-Thi KT Nguyen MD. The value of neutrophil-to-lymphocyte ratio, platelet-to-lymphocyte ratio, red cell distribution width, and their combination in predicting acute pancreatitis severity. *Eur Rev Med Pharmacol Sci*. (2023) 27:11464–71. 10.26355/eurrev_202312_34585 38095394

[49] Lu J Wang Z Mei W Peng K Zhang L Wang G et al. A systematic review of the epidemiology and risk factors for severity and recurrence of hypertriglyceridemia-induced acute pancreatitis. *BMC Gastroenterol*. (2025) 25:374. 10.1186/s12876-025-03954-4 40375154 PMC12082898

[50] Piñerúa-Gonsálvez JF Ruiz-Rebollo ML Fernández-Salazar L. Assessing the predictive value of the C-reactive protein and neutrophil-to-lymphocyte ratio combined score for organ failure in acute pancreatitis. *Arq Gastroenterol.* (2025) 62:e24119. 10.1590/S0004-2803.24612024-119 40531683 PMC12176416

[51] Huynh TM Tran A Tran DT Dao YTH Vo TDA. Novel classification and regression tree-driven decision tree combining neutrophil-to-lymphocyte ratio and C-reactive Protein for early prognostication of severe acute pancreatitis: a prospective Vietnamese cohort study. *Clin Transl Gastroenterol*. (2025) 16:e00919. 10.14309/ctg.0000000000000919 40924813 PMC12637335

[52] Isogai M. Pathophysiology of severe gallstone pancreatitis: a new paradigm. *World J Gastroenterol*. (2024) 30:614–23. 10.3748/wjg.v30.i7.614 38515949 PMC10950616

[53] Cheng S Zhang Y Jiang L Li J Jiang R Wang F. The association between inflammatory biomarkers and gallstones in Americans under 50 years old. *BMC Gastroenterol*. (2025) 25:393. 10.1186/s12876-025-03994-w 40399796 PMC12096754

